# Identification of Oxidative Stress in Red Blood Cells with Nanoscale Chemical Resolution by Infrared Nanospectroscopy

**DOI:** 10.3390/ijms19092582

**Published:** 2018-08-30

**Authors:** Francesco S. Ruggeri, Curtis Marcott, Simone Dinarelli, Giovanni Longo, Marco Girasole, Giovanni Dietler, Tuomas P. J. Knowles

**Affiliations:** 1 Department of Chemistry, Cambridge University, Cambridge CB21EW, UK; 2 Department of Materials Science and Engineering, University of Delaware, Newark, DE 19716, USA; marcott@lightlightsolutions.com; 3 Light Light Solutions, Athens, GA 30608, USA; 4 Institute of Structural Matter, ISM-CNR, via del Fosso del Cavaliere 100, 00133 Rome, Italy; simone.dinarelli@artov.ism.cnr.it (S.D.), longo@ism.cnr.it (G.L.), girasole@ism.cnr.it (M.G.); 5 Laboratoire de Physique de la Matière Vivante, Ecole Polytechnique Fédérale de Lausanne (EPFL), CH-1015 Lausanne, Switzerland; 6 Cavendish Laboratory, Department of Physics, University of Cambridge, Cambridge CB3 0HE, UK

**Keywords:** red blood cells, infrared nanospectroscopy, atomic force microscopy, oxidative stress, membrane peroxidation

## Abstract

During their lifespan, Red blood cells (RBC), due to their inability to self-replicate, undergo an ageing degradation phenomenon. This pathway, both in vitro and in vivo, consists of a series of chemical and morphological modifications, which include deviation from the biconcave cellular shape, oxidative stress, membrane peroxidation, lipid content decrease and uncoupling of the membrane-skeleton from the lipid bilayer. Here, we use the capabilities of atomic force microscopy based infrared nanospectroscopy (AFM-IR) to study and correlate, with nanoscale resolution, the morphological and chemical modifications that occur during the natural degradation of RBCs at the subcellular level. By using the tip of an AFM to detect the photothermal expansion of RBCs, it is possible to obtain nearly two orders of magnitude higher spatial resolution IR spectra, and absorbance images than can be obtained on diffraction-limited commercial Fourier-transform Infrared (FT-IR) microscopes. Using this approach, we demonstrate that we can identify localized sites of oxidative stress and membrane peroxidation on individual RBC, before the occurrence of neat morphological changes in the cellular shape.

## 1. Introduction

Red blood cells (RBCs), or erythrocytes, play a crucial role in the transport of oxygen via the circulatory system. Mature human RBCs are flexible biconcave disks without a nucleus [1,2,3,4,5,6]. They are roughly 8 µm in diameter, 1 µm thick at the rim and hundreds of nanometres thick in the central biconcavity. Compared to the usual complexity of nucleated cells, RBCs have unique characteristics, such as a simplified architecture and metabolism, exceptional mechanical properties and elasticity that are necessary to ensure their correct functioning in vivo. Indeed, erythrocytes are required to squeeze through capillaries with diameters in the sub-micrometre range and ensure oxygen delivery to all body tissues [3]. During its average life span in the cardiovascular system of approximately 120 days, a typical single RBC will travel about 240 km through veins, arteries, and narrow capillaries [3,4,5,7].

RBCs are simplified and specialized cells where mitochondria as well as nucleus and nucleic acids are absent. They can be considered as a sac containing haemoglobin (80% of RBC protein content), where the sac is the plasma membrane. The cytoskeleton mostly consists of a network of spectrin (10% of RBC protein content) and actin. This protein network is anchored to the overlying plasma membrane resulting in a peculiar structure, usually called membrane-skeleton, which is interconnected with the main activities related to the normal cellular function. In particular, the membrane-skeleton is responsible for maintaining the RBCs’ shape and the reversible deformations needed for their transport in capillaries [8,9,10]. As consequence of the interaction with the environment and of cell aging, an RBC undergoes severe chemical, morphological and mechanical changes (related to its state of health) that are mediated by alteration of its biochemical or biophysical status [11,12,13]. In particular, the lack of nucleic acids results in the inability to self-replicate and to implement a genetic response to the stimuli induced by the environment. The cell modification results in alteration of the plasma membrane, protein aggregation, skeleton detachment from the membrane and in the occurrence of lipid peroxidation that, as a whole, lead to the development of altered morphologies, such as spiky (echinocytes) or spherical (spherocytes) cells [14,15,16]. In these phenomena, a fundamental role is played by the oxidative stresses that are known to initiate or trigger degradative pattern at the base of metabolic or pathological anomalies [17,18,19]. In the case of RBCs, the presence of great amount of reactive oxygen species (ROS) is larger than usual due to the continuous oxygen flux across the membrane during their lifespan. The monitoring of cell oxidation is usually performed by measuring the biochemical consequences of ROS-induced injuries: lipid peroxidation and protein carbonylation [20]. For this reason, the enzymatic apparatus, that contrasts the intracellular peroxidation, is strengthened in these cells [21].

Atomic force microscopy (AFM) has emerged in the last decades as one of the most powerful and versatile single molecule techniques because of the possibility to acquire 3-dimensional (3-D) morphological maps of specimens on a surface [22]. In particular, AFM has been applied to investigate several aspects of the erythrocytes’ life, death and interaction with the environment by studying with nanoscale resolution their morphology and nanomechanics [1,2,14]. Conversely, infrared (IR) spectroscopy is a powerful tool for chemically characterizing materials, including biological samples and cells [23,24]. For studies of single cells, however, the diffraction-limited spatial resolution of commercial Fourier Transform Infrared (FT-IR) microspectrometers (3–10 µm) is insufficient to resolve features within a cell. The lack of spatial resolution has prevented the characterization and correlation of the chemical and structural properties of an individual RBC at the sub-cellular level. This technological gap has hampered the detailed investigation of the modification that individual RBCs undergoes during their aging or following oxidative stress at the nanoscale.

Recently, it has been shown that coupling an AFM to a tuneable IR laser source can provide a new tool, which combines the nanometer-scale spatial resolution of AFM with the chemical and structural analysis of IR spectroscopy [25]. This AFM-based infrared nanospectroscopy method (AFM-IR) is capable of producing local IR spectra, single wavenumber IR absorbance images, and contact resonance peak frequency stiffness images with nanoscale spatial resolution [26]. The pulsed and tuneable IR laser source illuminates an area of ~30 µm diameter of the sample; however, the spatial resolution advantage of AFM-IR over conventional FT-IR spectroscopy derives from the fact that the near-field aperture of the IR detector is nominally the AFM tip diameter, which is in the order of 10 nm [27,28]. Remarkably, for samples possessing a thickness smaller than 1–1.5 µm, the IR signal increases linearly with the sample thickness [29]. The spectra produced by scanning the tuneable laser source through the wavenumber range of interest are generally in perfect agreement with FT-IR transmission spectra. Thus, standard approaches and rules for interpreting IR spectra, including peak wavenumber shifts and band shape changes, apply to AFM-IR spectra in a similar manner [27]. Recent studies have firstly demonstrated the capability of AFM-IR to localize infectious agents in RBCs [30]. In addition, this approach has enabled the direct measurement and correlation of the morphological, chemical and secondary/quaternary structural properties of protein and for characterizing a wide range of systems in life science at the nanoscale [25,28]. More specifically, the method has excelled in studying the properties of heterogeneous samples, such as protein micro-droplets for drug delivery [31], the process of protein aggregation and amyloid formation [25,32,33,34,35], localization of glycogen structure with specific IR signature inside *Candida albicans* fungi cells [36], viruses inside infected bacteria [37], polymers inside *Rhodobacter capsulatus* cells [38], distribution of protein-rich material in *E. Coli* and human HeLa cells [39], presence of exogenous elements such as metal-carbonyl compounds in human cells, hair composition [40] and variations in the primate osteonal bone composition [41,42].

Here, we use AFM-IR nanospectroscopy to acquire nanoscale resolved IR spectra, morphology, chemical and stiffness 3-D maps of RBCs. Our aim is to correlate at the nanoscale the nanomechanical, structural and chemical properties of the sub-compartments of cells to identify the first signs of oxidative stress and membrane peroxidation. We demonstrate that infrared nanospectroscopy is capable of identifying the chemical signs of oxidative stress in RBCs, related to aging, before of the occurrence of a morphological change in their native biconcave cell structure.

## 2. Results

### 2.1. Nanoscale AFM-IR of Individual RBCs

In this study, we aimed at correlating with nanoscale chemical resolution the morphological and chemical modifications that RBCs undergo during aging by exploiting the capabilities of AFM-IR. Here, the ageing path of erythrocytes has been followed by monitoring the behaviour of the most typical morphologies that indicate the progress of cell senescence: namely, biconcave, spherocytes and echynocytes. Each of these morphological phenotypes characterize a given ageing time and results from a specific protein structure and cytoskeleton-to-membrane interactions [43].

**Figure 1:**
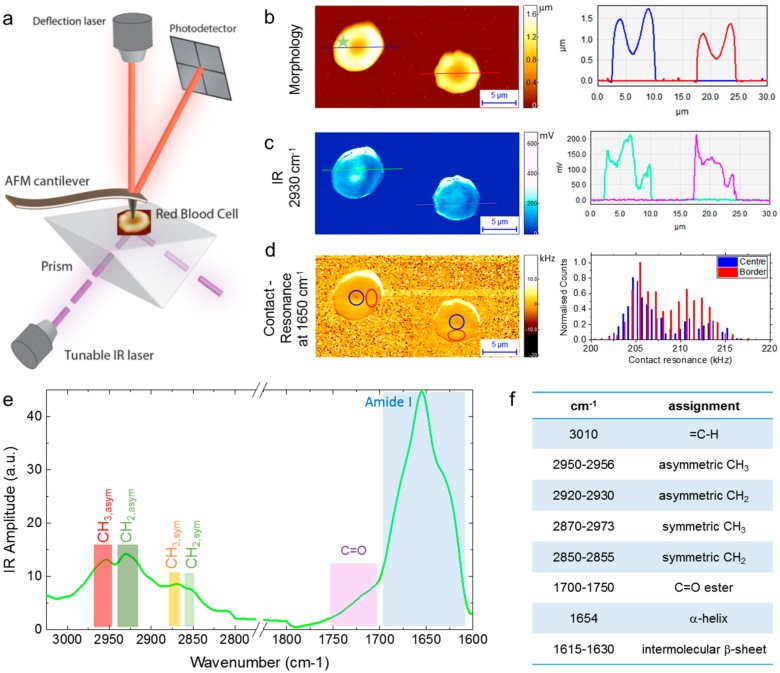
Infrared nanospectroscopy measurements of RBCs. (**a**) Schematic representation of the AFM-IR setup that enables the simultaneous acquisition of: 3-D (**b**) morphology map, with examples of cross-sectional height of the cells; (**c**) chemical map at 1650 cm^−1^, with examples of cross-sections of IR absorption of the cells acquired at the locations indicated by the light blue and pink lines; and (**d**) cantilever contact-resonance maps of individual cells, which are monotonically related to the cell stiffness; the histogram on the right represents the distribution of values of the tip-sample contact resonance within the red (border) and blue (centre) circles within the cells. (**e**) After acquisition of the maps is completed, the AFM tip can be positioned (green star in panel b) with nanoscale resolution (≈20–50 nm) on the cell to acquire an IR spectrum, averaged from 5 independent spectra. (**f**) We focused our spectroscopic investigation in the IR lipid region (2800–3050 cm^−1^) and amide band I, related to the secondary and quaternary structural conformation of proteins.

Figure 1a shows a schematic illustration of the AFM-IR setup used in this study. A tunable infrared laser is focused on the bottom of a ZnSe prism, where the beam is reflected by total internal reflection. At the interface of the reflection, an evanescent wave interacts with the sample on the substrate. If the wavenumber of the exciting laser radiation pulse matches one of the molecular vibrational energy transition levels of the sample, the light is absorbed. This absorption causes a thermal heating and expansion, which is detected with nanoscale resolution (≈10 nm) by the AFM tip in contact with the sample. At each cycle of the pulsed laser, the cantilever is kicked out of contact from the sample and it rings down at its natural resonant frequencies. The peak-to-peak amplitude of the oscillation is measured in real time by deflecting a second laser from the top surface of the cantilever. Performing a fast Fourier transform (FFT) on the raw cantilever deflection signal produces a plot of the ringdown responses, at the cantilever normal modes of vibration. The IR absorbance at each wavenumber is proportional to the peak-to-peak amplitude of the raw deflection signal or the peak amplitude, termed IR amplitude, of one of the individual ringdown frequencies in the FFT plot [25,27]. Multiple ringdown pulses can be co-added to improve the signal-to-noise ratio (S/N) before stepping to the next laser wavenumber. While acquiring morphology, IR absorption maps are obtained scanning in a raster way the AFM cantilever on the sample surface maintaining the laser wavelength fixed. Spectra are further obtained by sweeping the laser wavelength maintaining the position of the AFM cantilever fixed.

The system enables the simultaneous measurement of conventional morphology and the mapping of chemical and mechanical properties of the samples. RBCs are ideally suited for AFM-IR studies since, when dehydrated, their height is generally smaller than 1–1.3 µm, thus allowing a regime of linearity of the IR signal as a function of the thickness of the sample and of the intensity of the laser, which was kept constant for all the measurements [29,44]. This enables the study of the chemical heterogeneity of individual cells in the acquired IR maps at fixed wavenumber. In particular, in this work, we focused our attention on the antisymmetric methylene stretching (CH_2_ asymmetric) of lipids at 2930 cm^−1^, which can be related to lipids biophysical properties and chemical state within the cell membrane, and in particular to lipids structure, chain length, oxidative stress and membrane peroxidation [23,45,46,47,48]. In Figure 1b–d are shown three images of two RBCs dried onto the surface of a ZnSe substrate recorded simultaneously: (b) an AFM morphology 3-D map with the relative cross-sectional dimensions of the two cells; (c) an IR absorbance image and two cross-sections of the signal, recorded at the CH_2_ antisymmetric stretching wavenumber of lipids (2930 cm^−1^), where it its evident the internal chemical heterogeneity of the cells; and (d) a contact resonance peak frequency images (indicating the relative mechanical stiffness) of the two RBCs. The sample-tip contact resonance is monotonically correlated with the stiffness of the sample, where higher contact resonance is related to a higher stiffness [31,32]. A histogram distribution of the contact resonance in the center and at the border of the cells shows that the border is significantly stiffer than the centre, as observed previously (Figure 1d) [1,2,6]. Once the acquisition of the AFM-IR maps was completed, we placed the AFM tip on a specific location of the cells to acquire nanoscale resolved IR spectra in the ranges between 1400–1800 cm^−1^, with particular focus in the Amide I (1600–1700 cm^−1^, hereafter named the protein region) and C=O stretching (1700–1730 cm^−1^) vibrations, and 2800–3050 cm^−1^ (methylene and methyl symmetric and antisymmetric stretchings, hereafter named the lipid region) (Figure 1e). The positions of the main spectroscopic assignments used in this study are summarized in Figure 1f.

### 2.2. Sub-cellular Investigation of Individual RBCs

The morphology map in Figure 1b shows biconcave-shape cells, which indicates their healthy state. However, this conventional AFM topography map does not provide any insights into the biophysical properties of the cells. The IR absorbance image, on the other hand, shows a high degree of heterogeneity in the lipid-related signal (Figure 1c). In particular, the map at 2930 cm^−1^ shows a higher absorption at the centre of the cells and a lower contact-resonance frequency, which indicates that the cell is softer in the centre. Thus, we aimed at investigating the chemical differences between the central and border region of individual RBC with nanoscale resolution.

**Figure 2:**
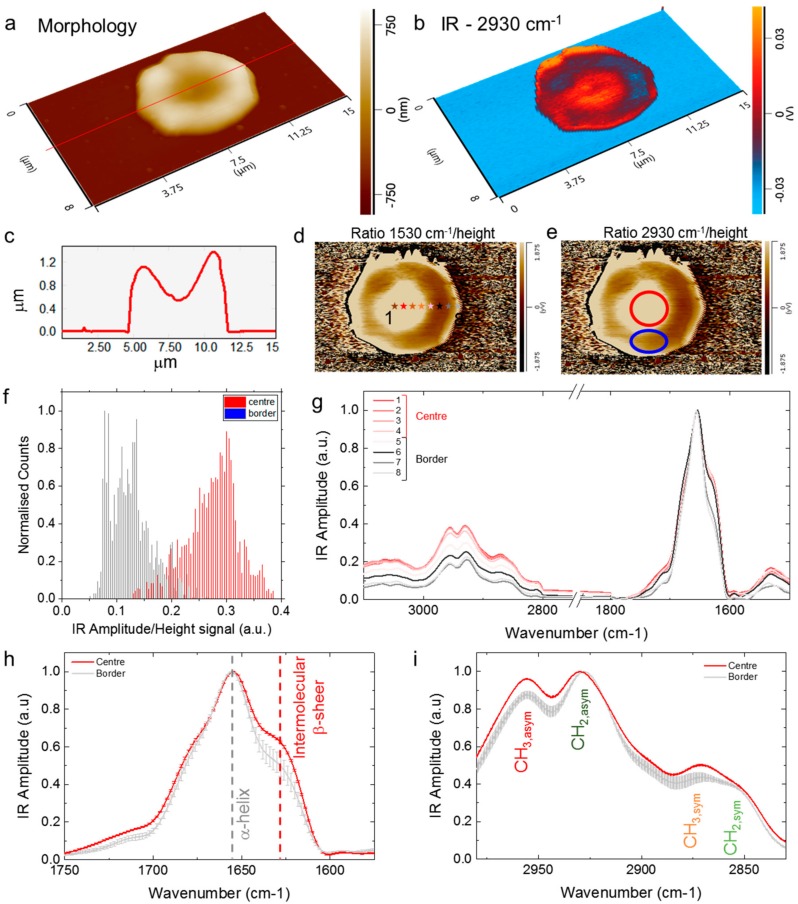
Subcellular chemical characterisation of an individual biconcave RBC. 3-D (**a**) morphology and (**b**) IR absorption maps of the cell. (**c**) Morphology cross-section. Ratio images obtained by the division of the IR signal at (**d**) 1530 cm^−1^ (amide II, protein) and (**e**) 2930 cm^−1^ (CH_2_ asymmetric stretching, lipids) showing a higher absorbance signal at the centre of the cell (red circle) than at the border (blue circle). (**f**) Histogram distribution of the IR signal in the ratio map at 2930 cm^−1^ in the central (red circle) and border (blue circle) region of the cell. (**g**) Average smoothed, but not normalised spectra acquired in the two regions of the cell (centre, violet to orange stars; border, grey scale stars; the spectrum at each position is the average of 5 spectra) confirm the higher IR absorption of the central region. Normalised and averaged spectra in the (**h**) Amide band I and the (**i**) lipid region with their standard deviation (8 different positions, *n* = 40 spectra, from the centre to the border of the cell).

In order to confirm the observed structural heterogeneity within a cell, one of the two biconcave RBCs in Figure 1 was examined in more detail and higher resolution (Figure 2). In Figure 2a,b, we show a 3-D representation of the morphology and IR absorption map of the cell at 2930 cm^−1^, corresponding to the antisymmetric methylene (CH_2_) stretching of lipids. The height of the cell ranges within 0.8–1.2 µm (Figure 2c), thus granting the linearity of the IR absorption signal as a function of the sample thickness. The chemical map shows a significant difference in absorption between the centre and the border of the cell, which show areas with markedly lower lipid absorption (dark areas in Figure 2b). In order to evaluate the spectroscopic behaviour independently from the sample thickness, we took advantage from the linearity of the signal and calculated the ratio maps between the IR absorption maps and the corresponding morphology map, both in the CH_2_ antisymmetric stretching region of lipids and the amide band II of proteins (Figure 2d,e). Both ratio maps confirm that the centre of the cell has a major IR amplitude absorption than the border, where there are dark regions corresponding to a lower IR absorption, as confirmed by a quantification of the IR signal in the absorption map at 2930 cm^−1^ (Figure 2f), indicating that the biophysical properties of the central and border regions of the cells are deeply different as independently proven by the different nanomechanical properties [1,2].

To further investigate the biophysical and spectroscopic differences between central and border regions of the cell, we acquired nanoscale resolved spectra at 500 nm intervals along the RBC, from locations indicated by the coloured stars in the maps (Figure 2d,g). Each spectrum derives from the average, smoothing and normalisation to one of at least five spectra acquired from each position (n > 40). In good agreement with the IR absorption and the ratio maps, the spectra at the centre of the cell have higher IR signal in the lipid region in the centre of the cell than in its border (Figure 2g). Then, we calculated the average of the spectra in the protein and lipid region and we normalised them to their maximum to compare their chemical properties. We focused our attention on the amide band I (Figure 2h) and the C-H stretching lipid region (Figure 2i). The protein region is related to the conformation of the proteins within the cell, and in particular quaternary and secondary structure conformation. While, the lipid region is intimately related to the structure and chemical state of lipids and cell membrane. The broad, intense amide I band has a peak wavenumber of 1654 cm^−1^, assigned as C=O stretching vibration and related to the α-helix secondary structure of the main protein component of the cell, which is hemoglobin. In the amide band, there is also visible a clear shoulder at approximately 1625 cm^−1^, which can be related to the intermolecular β-sheet content of the cytoskeleton of the cell. Remarkably, the shape of the amide I band shows that the right edge of the cell possesses a decreased content of intermolecular β-sheet secondary structure (Figure 2h). The lipid region shows methyl antisymmetric and symmetric stretching bands at 2955 and 2870 cm^−1^, respectively, while the more intense antisymmetric methylene band has a peak wavenumber of 2930 cm^−1^ (Figure 2i). The intensity ratio of the 2930 cm^−1^ (CH_2_ antisymmetric stretching) to 2955 cm^−1^ (CH_3_ antisymmetric stretching) bands is different between the central and border region of the cell. In particular, the intensity of the CH_2_ antisymmetric band 2930 cm^−1^ is significantly more intense relative to the 2955 cm^−1^ CH_3_ band at the border of the cell. This difference indicates there are longer lipid chains at the border of the cell than in its central region [23,47,48]. As well as previously reported [45,47,48], these data demonstrate that the edge of the cell is showing evidence of oxidative stress [23,46,47]. Indeed, the outer layer of the RBC is mechanically stiffer, has a higher concentration of long chain CH_2_ groups and has a lower content of intermolecular β-sheet structure, which suggests a weakening of the cell membrane-skeleton structure [21].

### 2.3. Comparison of Biconcave and Echinocyte

**Figure 3:**
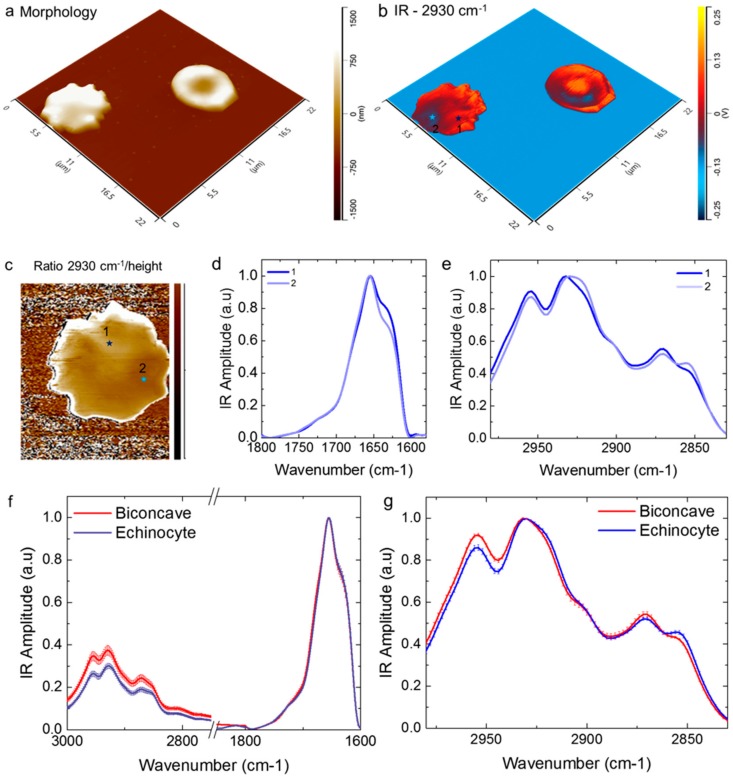
Chemical comparison of a biconcave and an echinocyte RBCs. 3-D (**a**) morphology and (**b**) IR absorption maps of the cells. (**c**) Ratio images detail of the echinocyte obtained by the division of the IR signal at 2930 cm^−1^ (CH2 asymmetric stretching, lipids) and the morphology map. Average smoothed and normalised spectra acquired in the spectroscopic (**d**) protein region and (**e**) lipid region for a bright (1) and dark (2) region of absorption within the echinocyte (each spectrum is the average of 5 independent spectra); the dark region of the cell shows sign of oxidative stress. (**f**,**g**) Averaged and normalised spectra within several dark (*n* = 45) and bright areas (*n* = 30) within the two cells in the amide band I and the lipid regions with their standard deviation.

After identifying the signs of oxidative stress within biconcave RBCs, we aimed at investigating if similar changes occur in RBCs with altered morphologies. In Figure 3b, AFM-IR morphology (Figure 3a) and chemical maps in the CH_2_ antisymmetric stretching (Figure 3b, 2930 cm^−1^) for biconcave and echinocyte RBCs are shown. Within both phenotypes, the chemical mapping shows a high heterogeneity of the IR absorption at 2930 cm^−1^, related to the CH_2_ antisymmetric band of lipids. The biconcave cell showed a lower lipid absorption at its edge, as observed above in Figure 2; however, the echinocyte cell had internal regions of higher and lower absorption that could not be correlated with any specific pattern (Figure 3c).

Next, we focused our attention on the echinocytes and we divided the IR signal by the morphological height in order to rule out the dependence of the signal from the cell thickness (Figure 3c). In the ratio image of the echinocyte cell, we could observe the presence of not evenly distributed dark and bright regions of IR absorption. Following the observation of a heterogeneous chemical response related to lipids, we acquire nanoscale localised IR spectra in the protein (Figure 3d) and lipid (Figure 3e) regions. The spectrum of the dark region (position 2, Figure 3d,e) showed a decreased content of intermolecular β-sheet and an increased ratio between the CH_2_ and CH_3_ antisymmetric bands when compared to the bright region of IR absorption (position 1, Figure 3d,e). The observed spectroscopic differences between the regions of the echinocyte cell indicated a different state of oxidation that correlates with the spectroscopic changes observed within the biconcave RBC (Figure 2). However, for these morphologically altered cells, it was also possible to observe a change in the ratio between the less intense CH_2_ and CH_3_ symmetric bands and a small but significant shift at lower wavenumber of the two CH_3_ bands, which can be related to a state of oxidative stress, as previously reported in literature [45,48]. These spectral differences demonstrated the presence of higher oxidative stress within the dark area of cell, as indicated by the presence of longer lipid chains and a likely degraded cell membrane-skeleton structure (Figure 3d,e).

After studying at the subcellular level the echinocytic cell oxidation state, we aimed at comparing its average chemical state with the biconcave cell on the right in Figure 3a,b. We acquired several spectra at random positions on the two cells (*n* = 35 and *n* = 45 for the two cells). Then, we smoothed, averaged and normalised the spectra with respect to the amide band I (Figure 3f). At first, it was possible to observe that the ratio between lipids and protein absorption differs for the two phenotypes. Indeed, the echinocyte had a significantly lower IR signal in the C-H lipid region of absorption than the biconcave one, which might be related to the lipid loss during membrane peroxidation in aged RBCs [1,4,46,48]. Furthermore, the observation of the lipid C-H region clearly showed that the echinocyte cell is on average more oxidated than the biconcave cell (Figure 3f). Indeed, the echinocyte cell showed, on average, an increased ratio between both the CH_2_ and CH_3_ symmetric and antisymmetric bands, which indicate oxidative stress and are related to the presence of longer lipid chains (Figure 3g) [23]. Remarkably, on average it was not possible to observe any significant difference between the two cells in the amide band I signal. Thus, at the average single cell level, for these two cells, it was not possible to infer any information regarding the different state of the membrane-skeleton structure (Figure 3f).

### 2.4. Membrane Peroxidation of RBCs

**Figure 4:**
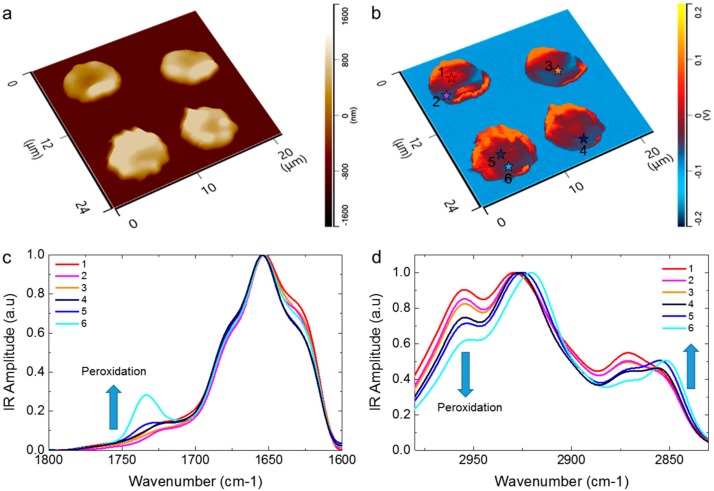
Membrane peroxidation of RBCs. 3-D (**a**) morphology and (**b**) IR absorption maps of the cells, where the dark region of the cell shows sign of oxidative stress. Average, smoothed and normalised spectra whose position is indicated by the coloured stars in the map (spectra from 6 positions, each one derived from the average of 5 independent spectra, *n* = 30) acquired in the spectroscopic (**c**) protein and (**d**) lipid regions for bright (1–3) and dark (4–6) regions of absorption within the cells. The ratio of methylene and methyl stretching directly correlate with the appearance of the ester C=O band, due to membrane peroxidation.

The observed similarities of the oxidative stress modifications between biconcave and echinocyte RBCs suggested that these modifications could further lead to membrane peroxidation, which is the typical hallmark of RBCs cellular aging. Figure 4a,b, shows a 3-D representation of the morphology and IR absorption maps of RBC deviating from the native biconcave shape. In particular, the two cells on the top of the images showed an expansion of their volume at their edge, while the two cells on the bottom had a spiky morphology. As observed in the previous examined cells (Figure 1, Figure 2 and Figure 3), we observed a high heterogeneity of the IR absorption at 2930 cm^−1^. In order to analyse the chemical state of the cells and in particular any possible membrane peroxidation, several spectra were acquired from different locations in the dark areas of IR absorption of biconcave and echinocyte cells (coloured stars, *n* = 30, in Figure 4a,b). The spectra were acquired in the protein and lipid regions and then averaged, smoothed and normalised (Figure 4c,d). The biconcave cells (position 1–3, Figure 4b) showed a spectral signature similar to that of previously analysed cells (Figure 1, Figure 2 and Figure 3). On the contrary, the echinocyte evidenced a higher methylene to methyl intensity ratio, indicating they possess longer chain hydrocarbons and higher oxidative stress at the locations with lower IR absorption examined in these RBCs (position 4–6, Figure 4b). In particular, the spectra collected at positions 5 and 6 showed evidence of a clear signal of membrane peroxidation, as demonstrated by the appearance of the ester carbonyl stretching (C=O) band at 1734 cm^−1^. Remarkably, the appearance of the ester C=O band directly correlated with a continuous change of the ratio between the intensities of both symmetric and antisymmetric methylene and methyl stretching, which are related to oxidative stress and lipid chain length. In addition, the spectra in position 4 to 6, also showed a net shift of the CH_2_ bands to lower wavenumber, suggesting that the lipid chains are more ordered than in any of the other spectra. Moreover, in average, the spectra showed a lower IR signal assignable to the spectroscopic signature of intermolecular β-sheet content than the spectra collected on the biconcave cells (position 1–3).

Altogether, the data in Figure 4 indicate that the early chemical modifications observed in the protein and lipid regions of spectra acquired on biconcave RBCs (Figure 1, Figure 2 and Figure 3) can ultimately lead to membrane peroxidation and deviation from the native biconcave morphology of the cells, which is likely play a role in inducing the echinocyte morphology. In addition, the oxidative state of the cell and its membrane peroxidation directly correlate at the sub-cellular scale with a loss of intermolecular β-sheet structure. Thus, oxidative stress and membrane peroxidation could be related to a degradation of the membrane-skeleton network.

## 3. Discussion

In this work, we exploit the capabilities of AFM-IR to demonstrate the possibility of acquiring nanoscale resolved chemical and mechanical maps onto erythrocytes. This approach has enabled the localization of oxidative stress and membrane peroxidation at the sub-cellular scale in healthy biconcave cells, before any morphological change related to oxidative stress occurred. In particular, we quantified the degree of oxidative stress within an individual RBC as a function of the relative amounts and in the molecular order of long chain CH_2_ groups. The difference in lipid chains lengths correlated with the nanomechanical properties of sub-compartments within an individual RBC, indeed the border of biconcave erythrocytes was stiffer and had longer lipid chains than its centre; these biophysical properties can be correlated to different composition and chemical state of lipids within the cell, such as their fluidity. Furthermore, we could correlate a higher oxidative stress to lower content of intermolecular β-sheet structure. These spectroscopic changes could be related to the membrane-skeleton integrity of the cells, through a mechanism that involves a rearrangement of the protein 4.1, which is the most important cytoskeleton protein with a significant β-sheet content in RBCs. Furthermore, our data suggest that in biconcave erythrocytes the external edge might be the region of the cell, which is more susceptible to oxidative stress. When compared to biconcave cells, echinocytes show an even lower IR absorption in the spectroscopic region of the C-H stretching of lipids and of intermolecular β-sheet structure. The early chemical changes at the border of biconcave cells led directly to the appearance of the ester carbonyl stretching (C=O) band at 1734 cm^−1^ in echinocyte demonstrating, at the nanoscale, the presence of membrane peroxidation. These observations suggest that the early chemical modifications observed at the border of biconcave cells are continuously ongoing during cellular aging. In this regard, it has been previously found that cell aging and oxidative stress in RBCs correlates with general morphological changes in the cellular shape and in the membrane roughness. Since in this work we observe variation in the β-sheet content also within healthy normal shaped cells, we demonstrate that the detection of membrane peroxidation and membrane-skeleton alterations is possible before a clear morphological change of the cellular structure has occurred. These latter data are particularly interesting in the light of recent findings evidencing that the morphological changes actually occur as consequence of environmental stimuli that act by modulating the metabolic pathways with the mediation of oxidative stresses [11]. The approach developed in this work can be very promising in order to improve our understanding of RBCs viability and function, which can be very important for the development of new treatments or the screening of pathologies at early stage of development, as well as to improve the methodology of blood conservation.

## 4. Materials and Methods

### 4.1. RBC Sample Preparation

Blood samples were obtained from healthy donors as previously described [49]. Briefly after venipuncture the blood was immediately diluted threefold with an isotonic pH 7.35 buffer solution (140 mM NaCl, 10 mM Potassium Phosphate, containing 1 mM EDTA disodium salt as anticoagulant). Three centrifugation cycles were performed (12 min each at 3200 rpm at 4 °C) to eliminate plasma enriched supernatant and the leukocytes layer. RBCs were re-suspended and washed four times under sterile conditions in glucose-free PBS medium (potassium phosphate 4 mM, EDTA 1 mM, and NaCl 140 mM), adjusted with NaOH to pH 7.35. To avoid proteolytic degradation, a protease inhibitor (phenylmethylsulfonyl fluoride, 1 mM) was added. After purification of erythrocytes from, approximately, 2 mL of blood, the cells were re-suspended in a 20% hematocrit. However, the smears on the substrate for the infrared nanospectroscopy investigation required the addition of 3 µL of plasma to 2 µL of the RBC suspension, in order to protect the erythrocytes and avoid cell-cell adhesion during the smear. Thus, the smears were performed with a 7–8% hematocrit, which is an optimal dilution to obtain a good number of properly spaced cells across the entire substrate.

### 4.2. AFM-IR Measurements

Samples for AFM-IR analysis were prepared by spreading a RBC suspension diluted in plasma (ratio 1:5) onto the surface of a ZnSe prism (internal reflection element, or IRE) and allowing the cells to dry in air. A number *n* = 25 of red blood cells were then examined using a nanoIR™ microspectroscopy system (Anasys Instruments, Santa Barbara, CA, USA) to support the conclusions of the manuscript. We used a scan rate of 0.02 Hz per line in contact mode. A silicon AFM cantilever (AppNano, CA, USA) with a nominal tip radius of 10 nm and an elastic constant of ~0.5 Nm^−1^ was used for all measurements. All images have a resolution of at least 500 × 100 pixels. All AFM height, IR absorbance amplitude, and contact resonance peak frequency images were first-order flattened using Analysis Studio software (Anasys Instruments, Santa Barbara, CA, USA). Statistical and cross-sectional analysis was performed by SPIP (Image metrology, Hørsholm, Denmark) and Origin Pro (OriginLab, Northampton, MA, USA) softwares.

The AFM-IR spectra were collected with a data point spacing of 2 cm^−1^ and 256 ringdown co-averages at each wavenumber position over the spectral range 1400–3100 cm^−1^. We used an OPO pulsed laser with a pulse width of 10 ns. The laser is tunable in the range of 1000–3300 cm^−1^ and in this range had a power between 2–7 mW (8% of OPO laser power). The spectral resolution is determined by the natural linewidth of the laser and is about 4–8 cm^−1^ over this range. All AFM-IR spectra were smoothed by a Fourier Transform filter and Savitzky-Golay filter (second order, seven points) using Origin Pro software (OriginLab, Northampton, MA, USA). The peak locations were determined using a three-point centre of mass method, rounded to the nearest wavenumber, and plotted from Essential FT-IR software (Operant). The experiments were performed at room temperature.
